# A pan-KRAS degrader for the treatment of KRAS-mutant cancers

**DOI:** 10.1038/s41421-024-00699-4

**Published:** 2024-06-28

**Authors:** Jie Yang, Qiao-Li Wang, Guan-Nan Wang, Jia-Cong Ye, Zi-Qian Li, Jing-Yun Wang, Zhao-Hui Liang, Shu-Xin Li, Cong Sun, Wen-Ting Liao, Yi-Jun Gao, Jing Wang, Yong Mao, Chunjing Yu, Guo-Kai Feng, Mu-Sheng Zeng

**Affiliations:** 1 State Key Laboratory of Oncology in South China, Guangdong Provincial Clinical Research Center for Cancer, Sun Yat-sen University Cancer Center, Guangzhou, Guangdong China; 2 Department of Oncology, Affiliated Hospital of Jiangnan University, Wuxi, Jiangsu China; 3 Department of Nuclear Medicine, Affiliated Hospital of Jiangnan University, Wuxi, Jiangsu China

**Keywords:** Proteolysis, Targeted therapies

## Abstract

*KRAS* mutations are highly prevalent in a wide range of lethal cancers, and these mutant forms of KRAS play a crucial role in driving cancer progression and conferring resistance to treatment. While there have been advancements in the development of small molecules to target specific KRAS mutants, the presence of undruggable mutants and the emergence of secondary mutations continue to pose challenges in the clinical treatment of KRAS-mutant cancers. In this study, we developed a novel molecular tool called tumor-targeting KRAS degrader (TKD) that effectively targets a wide range of KRAS mutants. TKD is composed of a KRAS-binding nanobody, a cell-penetrating peptide selectively targeting cancer cells, and a lysosome-binding motif. Our data revealed that TKD selectively binds to KRAS in cancer cells and effectively induces KRAS degradation via a lysosome-dependent process. Functionally, TKD suppresses tumor growth with no obvious side effects and enhances the antitumor effects of PD-1 antibody and cetuximab. This study not only provides a strategy for developing drugs targeting “undruggable” proteins but also reveals that TKD is a promising therapeutic for treating KRAS-mutant cancers.

## Introduction

It is estimated that ~20% of all human tumors have mutations in one of the *RAS* genes, among which *KRAS* is the most frequently mutated oncogene^1^. Lung adenocarcinoma (LUAD), pancreatic ductal adenocarcinoma (PDAC), and colorectal cancer (CRC) are highly lethal and exhibit high frequencies of *KRAS* mutations, with mutation rates of 32%, 86%, and 41%, respectively. The majority of *KRAS* mutations occur in codons 12, 13, 61, and 146, with mutations in codons 12 and 13 accounting for 90% of all *KRAS* mutations^2,3^. KRAS mutants induce constitutive activation of downstream signaling cascades^4^, which not only contributes to tumor development but also weakens or even abolishes the inhibitory effect of drugs targeting upstream effectors, such as epidermal growth factor receptor (EGFR)^5,6^. Furthermore, KRAS mutants repress the expression of interferon regulatory factor 2 (IRF2), thus inducing the resistance of cancers to immunotherapy^7,8^.

KRAS has long been considered an “undruggable” protein due to its spherical structure, strong affinity to GTP, and frequent mutations. These characteristics pose challenges for the development of targeted drugs using traditional strategies^9^. However, recent advancements have identified a binding pocket (S-IIP) in the KRAS G12C mutant that can be targeted by small molecules. Inhibitors such as sotorasib and adagrasib have been shown to interact with this pocket through covalent interactions, effectively suppressing the growth of G12C mutant non-small cell lung cancer (NSCLC)^10,11^. Additionally, a selective KRAS G12D inhibitor called MRTX1133 can also bind to the S-IIP pocket, but its interaction with KRAS is noncovalent^12^. Another small-molecule inhibitor, which acts by blocking the nucleotide exchange between KRAS-bound GTP and KRAS-bound GDP, has demonstrated broad inhibition of KRAS activity; however, its effectiveness against KRAS mutants with impaired hydrolytic activity, such as KRAS G12R and Q61L/R/K, is limited^13^. It is worth noting that the binding sites of these inhibitors are specific to particular KRAS mutants or a class of mutants. Moreover, the extreme inhibition of KRAS activity by inhibitors often leads to the emergence of unpredictable secondary mutations. Therefore, alternative strategies are necessary to develop pan-KRAS targeted drugs that can overcome these limitations^14,15^.

Disrupting the expression of the *KRAS* gene is an attractive strategy for abolishing the oncogenic effects of KRAS mutations. Theoretically, both DNA editing and RNA interference can decrease KRAS expression, but the operating conditions and off-target effects of these strategies have limited their clinical use thus far^16,17^. Selective protein degradation is another promising strategy for reducing KRAS in cancer cells^18^. One technique that has been developed for this purpose is proteolysis targeting chimeras (PROTACs). A PROTAC consists of two segments: a substrate or ligand for the E3 ubiquitin ligase and a ligand for the target protein. These segments are covalently linked through a linker. By recruiting the target protein to the E3 ligase, PROTACs facilitate its degradation in a proteasome-dependent manner^19–21^. Previous studies have demonstrated successful degradation of the KRAS G12C mutant using this approach^22,23^. Another attractive design of a protein degrader is Trim-Away, which takes advantage of the natural affinity of the E3 ubiquitin ligase TRIM21 to the Fc segment of a normal antibody. Trim-Away recruits the antibody–target protein complex to TRIM21 and thus induces the ubiquitination and subsequent proteasome-dependent degradation of the target protein^24^. The functional mechanism of Trim-Away suggests that a KRAS degrader can be constructed based on an antibody that has a strong affinity towards KRAS. Additionally, AdPROM is another antibody-based protein degrader that involves fusing the von Hippel‒Lindau (VHL) protein, which recruits the CUL2–RING E3 ligase complex, with a high-affinity binder for the target protein. AdPROM is introduced into targeted cells using a retrovirus vector^25^. The knocked-in green fluorescent protein (GFP)-tagged KRAS protein could be successfully degraded using GFP nanobody-based AdPROM^26^. When the GFP nanobody was replaced by an HRAS monobody, the degrader also induced a strong reduction in endogenous untagged KRAS, HRAS and NRAS, indicating that monobody-based degradation is not specific for KRAS^26^. These findings demonstrated the feasibility of using an antibody-based approach for designing targeted drugs against KRAS. Theoretically, the antibody-based KRAS degrader can induce the degradation of pan-KRAS mutants because the binding of antibody and antigen is not confined to a specific site.

Protein-based targeted drugs such as cetuximab and pembrolizumab have been widely applied for cancer therapy, but almost all protein-based drugs work in the extracellular space^27,28^. To successfully enter cells and inhibit activities of intracellular targets, the size of protein-based drugs should be small, the structure should be uncomplicated, and an effective cell-penetrating peptide (CPP) is very helpful^29^. Nanobody is the variable domain of an antibody’s heavy chain derived from alpaca, which has a mass of ~15 kD, much smaller than traditional antibodies ( ~150 kD); thus, nanobodies can easily penetrate various tissues and can be produced using prokaryotic or eukaryotic protein expression systems^30^. Nanobodies are therefore an ideal platform for the construction of novel target protein degraders.

Previous studies have demonstrated that the nontoxic peptide BR2 derived from buforin IIb can selectively recognize and enter cancer cells rapidly and specifically^31–33^. Moreover, the CTM peptide that can be recognized and bound by lysosomes has been used for inducing the selective degradation of target proteins^34,35^. Therefore, we have taken an approach different from the selective protein degraders currently in use and constructed a novel KRAS degrader based on a nanobody targeting KRAS and named it tumor-targeting KRAS degrader (TKD). TKD contains three elements: the cancer cell-penetrating peptide BR2, a nanobody that binds to KRAS, and the lysosome-recognizing and -binding motif CTM. We investigated the effect of TKD in degrading KRAS mutants and suppressing tumor growth. Considering that KRAS mutation is believed to be a potent driver of resistance to immunotherapy and targeted therapy, we further evaluated the role of TKD in sensitizing cancers to programmed cell death protein 1 (PD-1) antibody and cetuximab.

## Results

### TKD is designed based on the KRAS nanobody and shows a strong affinity to KRAS

**Fig. 1:**
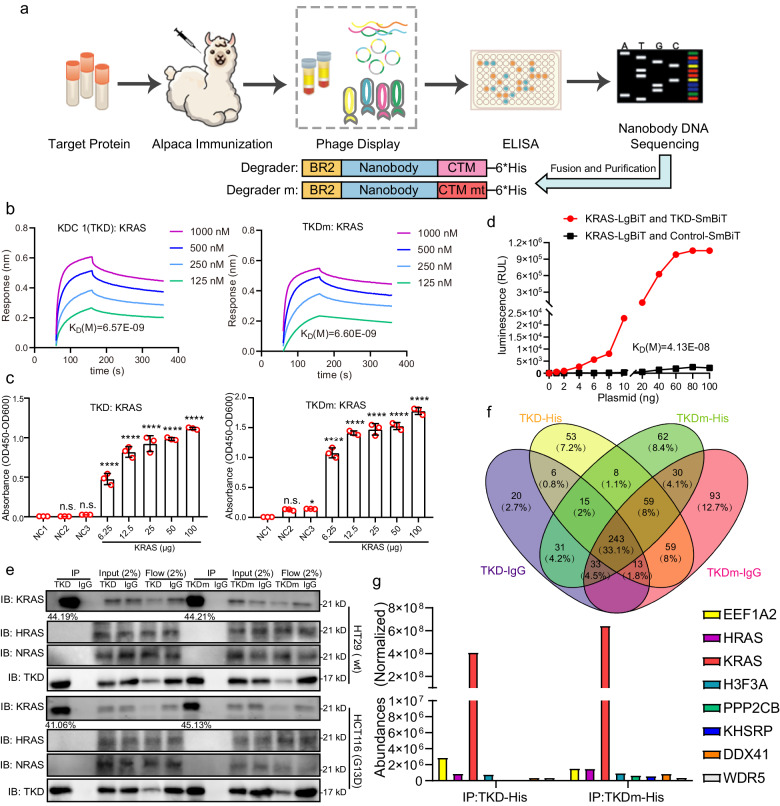
TKD interacts with KRAS with a strong affinity. **a** Workflow of the selective protein degrader construction. **b** BLI assays were performed using purified KRAS together with KDC 1 (TKD) or TKDm. The concentrations of TKD or TKDm flowing through the KRAS-coated probes are indicated. **c** KRAS, TKD, and TKDm were expressed and purified, and ELISA assays were performed to determine the affinity of KRAS to TKD or TKDm. NC1, empty control; NC2, TKD/TKDm + IgG control; NC3, IgG + KRAS (100 μg) control. **d** 293T cells were transiently co-transfected with KRAS-LgBiT and TKD (nanobody)-SmBiT, or control-SmBiT plasmids in different concentrations as indicated for 24 h, then the cells were incubated with substrate and the fluorescence intensity was measured to estimate the affinity of KRAS and TKD. **e** HT29 and HCT116 cells were treated with a combination of TKD or TKDm (0.5 μM) and Lys05 (10 μM) for 6 h and IP assays were performed by using a 6*His-tag antibody. The levels of KRAS/HRAS/NRAS and TKD/TKDm-His in the immunoprecipitants, the whole cell lysates (2% Input), as well as lysates flowing through the beads (2% Flow), were detected by western blotting assay, and the ratio of precipitated KRAS in total KRAS was analyzed. **f**, **g** The immunoprecipitants of HCT116 cells in **e** were collected for DDA MS analysis, the identified proteins (**f**) and their abundances (**g**) were analyzed. Statistical analyses were performed using two-way ANOVA; Error bars, SD; n.s. not significant; *P < 0.05; ****P < 0.0001.

To assess the efficacy of nanobody-based protein degraders in targeting intracellular proteins, we developed a protein degradation tool using the GFP nanobody. Initially, we constructed a fusion protein consisting of the peptide BR2^31^, a GFP nanobody^36^ and CTM^34^. The DNA sequence encoding the fusion protein was then cloned and inserted into an expression vector, and the fusion protein was produced in *Escherichia coli* to generate the GFP degrader (GD). We subsequently evaluated the activity of GD in degrading exogenously expressed GFP in the HCT116 CRC cell line. A negative control GDm was designed and purified, which contained a mutant CTM peptide (CTM mt) that cannot be recognized by or bound to lysosomes (Fig. 1a). To demonstrate the interaction of GD and intracellular GFP, we treated HCT116-GFP cells with 0.5 μM GD or GDm in the presence of 10 μM lysosome inhibitor Lys05. Subsequent co-immunoprecipitation (co-IP) and western blotting assays revealed that both GD and GDm interacted with GFP (Supplementary Fig. S1a). Fluorescence microscopy analysis showed that GD significantly reduced the fluorescence intensity of HCT116-GFP cells, whereas GDm treatment did not (Supplementary Fig. S1b). We then labeled the lysosome marker LAMP2 with an appropriate fluorescent antibody and performed immunofluorescence (IF) assays, which revealed that GFP accumulated in lysosomes when HCT116-GFP cells were treated with a combination of GD and Lys05 (Supplementary Fig. S1c). Additionally, the western blotting assay verified that the level of GFP reduced by GD could be blocked by Lys05 and another lysosome inhibitor, bafilomycin A1 (Baf-A1) (Supplementary Fig. S1d). These data demonstrate that GD induces lysosome-dependent protein degradation of GFP.

Encouraged by the success of the induced GFP degradation, we then constructed a similar tool for inducing the degradation of intracellular KRAS (Fig. 1a). We first used KRAS (1–169 amino acids (aa)) to immunize the alpaca to generate nanobodies, and the nanobody genes were then amplified and cloned and inserted into phages. Enzyme-linked immunosorbent assays (ELISAs) were performed twice to identify phages that had high affinity to KRAS, and 6 positive phages were identified and sequenced (Supplementary Table S1). Subsequently, different nanobody-encoding genes of these phages were fused with DNA fragments encoding BR2 and CTM to produce KRAS degrader candidates (KDCs). The affinity of different KDCs to KRAS was evaluated by biolayer interferometry (BLI) assays, and the data showed that the dissociation constant (*K*_D_ = 6.57E−09 M) between KDC 1 and KRAS was the strongest among the 6 KDCs (KDCs 2–6) (Fig. 1b and Supplementary Fig. S2a). Therefore, we chose KDC 1 as the potential KRAS degrader and named it TKD. We then constructed a corresponding negative control TKDm that has the same BR2 and nanobody sequence as TKD but contains a mutant CTM peptide. The BLI assay demonstrated that TKDm also possessed a similar affinity to KRAS (*K*_D_ = 6.60E−09 M) (Fig. 1b). In addition, ELISAs performed using purified TKD/TKDm and KRAS further confirmed that both TKD and TKDm bound to KRAS with high affinity (Fig. 1c). We further investigated the binding activity of TKD (nanobody) and KRAS in living cells using nanoLuc binary technology (NanoBiT). The data showed that after excluding non-specific binding caused by gene overexpression, TKD (nanobody) and KRAS began to exhibit interaction at very low plasmid transfection (1 ng), indicating that TKD maintained high KRAS-binding activity in living cells (Fig. 1d).

Then, we conducted the BLI assays using purified HRAS and NRAS, two proteins that have high similarity to KRAS, to determine the specificity of TKD to KRAS. Interestingly, our results showed that the response of TKD to HRAS or NRAS is very low, which means that there is no binding activity between TKD and HRAS/NRAS (Supplementary Fig. S2b, c). Besides, co-IP assays performed using lysates from HT29 (KRAS wildtype (wt)) and HCT116 (KRAS G13D) cells cotreated with Lys05 and TKD or TKDm showed that ~41%–45% of the intracellular KRAS was immunoprecipitated by TKD and TKDm, but almost no HRAS and NRAS were identified in the immunoprecipitants (Fig. 1e and Supplementary Fig. S2d). To further clarify the binding specificity of TKD to KRAS in living cells, the immunoprecipitants of TKD- and TKDm-treated HCT116 cells were further determined by Data-Dependent Acquisition (DDA) mode mass spectrometry (MS) analysis. Among the 8 proteins identified in both TKD and TKDm immunoprecipitants, KRAS possessed the highest abundance than any other proteins, further indicating that TKD has a specific binding affinity to KRAS (Fig. 1f, g). In summary, we constructed a KRAS nanobody-based degrader (TKD) that has a very specific and high affinity for KRAS.

### TKD targets cancer cells and induces KRAS degradation in a lysosome-dependent manner

**Fig. 2:**
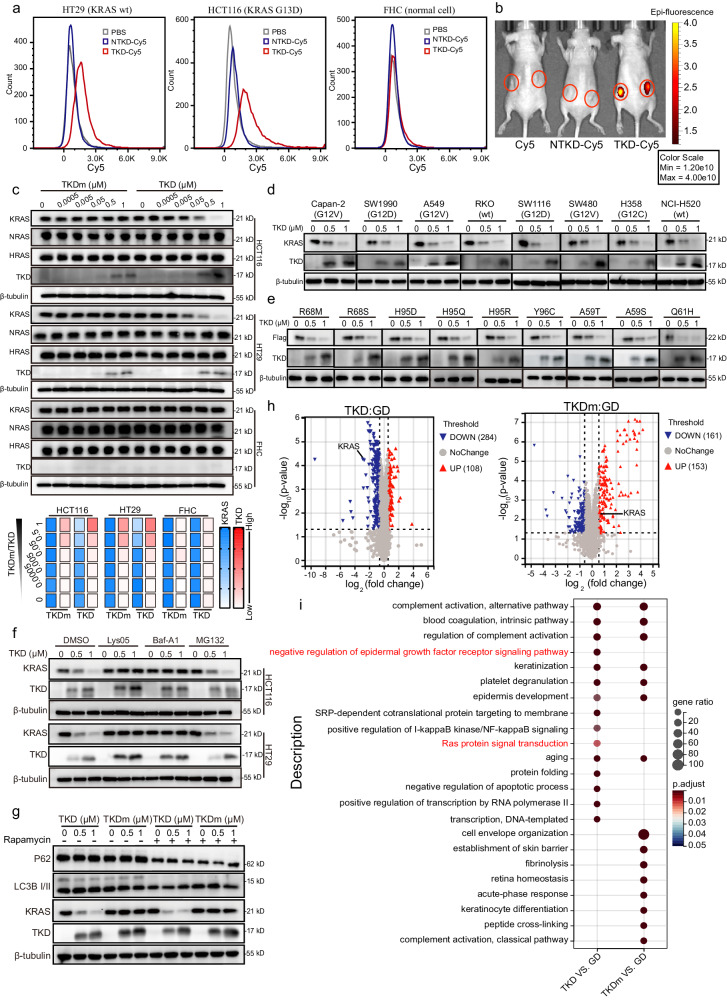
TKD specifically targets cancer cells and induces lysosome-dependent KRAS degradation. **a** FHC, HT29 and HCT116 cells were treated with either PBS, TKD-Cy5 or NTKD-Cy5 for 2 h, and were then determined by flow cytometric analysis. NTKD, a fusion protein control lacking cell-penetrating peptide BR2 compared with TKD. **b** Cy5 and NTKD-Cy5 controls (100 μL, 60 μM), as well as TKD-Cy5 (100 μL, 60 μM) were injected into mice bearing HT29 cell (left flank)- and HCT116 cell (right flank)-derived tumors through tail vein, and the Cy5 fluorescence intensity was measured 2.5 h after the injection (*n* = 3). Red circle, tumor. **c** HT29, HCT116, and FHC cells were treated with TKD or TKDm at a series of concentrations as indicated for 24 h, and the expression of KRAS, HRAS, NRAS, TKD-His, and β-tubulin was detected. **d** Cancer cells with different KRAS mutants as indicated were treated with TKD for 24 h. The expression of KRAS, TKD-His, and β-tubulin was detected. **e** HT29 cells stably expressing KRAS G12C combined with the secondary mutations as indicated were treated with TKD for 24 h. The expression of Flag, TKD-His, and β-tubulin was detected. **f** HT29 and HCT116 cells were treated with TKD alone or together with Lys05 (10 μM), Baf-A1 (100 nM), MG132 (1 μM) for 24 h. The expression of KRAS, TKD-His, and β-tubulin was detected. **g** H358 cells were treated with TKD alone or a combination of TKD and rapamycin (25 μM) for 24 h, then cells were lysed and the expression of LC3B I/II, P62, KRAS, TKD-His, and β-tubulin were determined using western blotting assays. **h** TKD- or TKDm-treated HCT116 cells were lysed for quantitative proteomic analysis, and the differential expression proteins were analyzed. **i** The biological processes of differential expression proteins in TKD- and TKDm-treated HCT116 cells were enriched using Gene Ontology (GO) analysis.

Considering the high affinity and specificity of TKD toward KRAS, we then evaluated the effect of TKD on KRAS degradation in cancer cells. TKD was purified and characterized by using Ni-NTA resin, ion exchange chromatography (IEC), and high-performance liquid chromatography (HPLC), and the residual endotoxin was examined. The data showed that the TKD used for in vitro and in vivo experiments featured high purity and little endotoxin (Supplementary Fig. S3a–d). To determine the cancer cell-targeting ability of TKD, we treated the normal colon epithelial cell line FHC and the CRC cell lines HT29 and HCT116 with Cy5-labeled TKD (TKD-Cy5) for 2 h. Flow cytometric analysis demonstrated that both HT29 and HCT116 cells were stained with TKD-Cy5, whereas FHC was not (Fig. 2a). We further determined the tumor-targeting ability of TKD in mice bearing HT29- and HCT116-derived tumors. Live imaging analysis showed that TKD-Cy5 accumulated rapidly in the tumors ~2.5 h after tail vein injection, whereas the Cy5 and NTKD-Cy5 controls did not, indicating that TKD has a strong tumor-targeting ability in vivo (Fig. 2b). Then, we treated HT29, HCT116, and FHC cells with different doses of TKD or TKDm. Western blotting assays showed that KRAS was reduced by TKD in a dose-dependent manner in cancer cells but not in normal FHC cells or TKDm-treated cells. Furthermore, the expression of the other two members of the RAS family, namely, HRAS and NRAS, remained stable during TKD treatment (Fig. 2c and Supplementary Fig. S3e). Besides, TKD induced broad degradation of KRAS in different cancer cells that harbor different KRAS mutants (Fig. 2d and Supplementary Fig. S3f). Importantly, previous studies demonstrated that the secondary mutations of *KRAS*, such as Q61H, R68M/S, H95D/Q/R, Y96C, A59S, and A59T, are the main cause of clinical resistance to approved KRAS G12C inhibitors, such as adagrasib and sotorasib^37^. To determine the inhibitory effect of TKD on these secondary mutants, we modified HT29 cells using lentivirus that stably expressed KRAS with the above secondary mutations. The western blotting results showed that all mutants were successfully degraded after treatment with TKD, suggesting that the secondary *KRAS* mutations could not lead to resistance to TKD (Fig. 2e and Supplementary Fig. S3g).

Importantly, western blotting assays demonstrated that KRAS degradation induced by TKD was blocked in cancer cells by Lys05 and Baf-A1, but not by MG132, a proteosome inhibitor, further supporting that TKD promotes KRAS degradation in a lysosome-dependent manner (Fig. 2f and Supplementary Fig. S3h). Of note, the treatment of TKD did not affect the activity of lysosomes, indicating that TKD-induced KRAS degradation does not disrupt normal lysosomal functions in cells (Fig. 2g and Supplementary Fig. S3i). Besides, quantitative proteomic analysis showed that compared with TKDm, TKD significantly reduced the expression of KRAS in cells (Fig. 2h). After enrichment analysis of all differentially expressed proteins caused by TKD, it was found that many of these proteins were enriched in KRAS-related signaling pathways such as EGFR regulation and RAS signal transduction, which further proved the specificity of KRAS degradation induced by TKD (Fig. 2i).

Overall, our data show that TKD is a tumor-targeting molecule that has a powerful ability to induce the degradation of different KRAS mutants, including secondary mutants, via lysosomes.

### TKD has good pharmacokinetic characteristics and few side effects

**Fig. 3:**
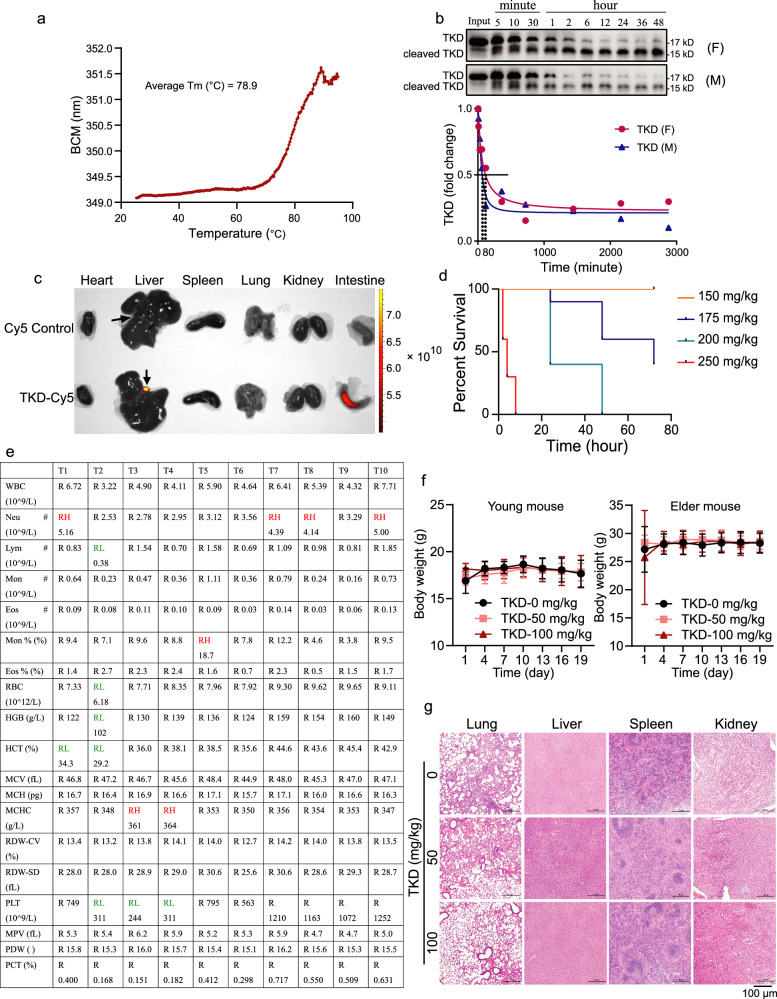
TKD shows a typical kinetics of protein drug metabolism and little side effects. **a** Protein stability analysis performed with 1 μg/μL TKD using Uncle (Unchained Labs, USA). Tm, denaturation temperature; BCM, centroid wavelengths of protein endogenous fluorescence emission spectra. **b** 0.1 μg purified TKD was mixed with 27 μL of human sera and incubated at 37 °C for different durations as indicated, the degradation of TKD was evaluated by western blotting assays, and the degradation curve was drawn using nonlinear regression based on the gray value. Input, purified TKD; F, sera derived from the female; M, sera derived from the male. **c** Main organs from mice treated with 60 μM Cy5 control or TKD-Cy5 through tail vein injection (2 h) were collected for imaging. *n* = 3; black arrow, gallbladder. **d** Mice treated with different doses of TKD, and the percent survival of each group was analyzed. *n* = 10. **e** Routine blood test of mice treated with 100 mg/kg TKD. *n* = 10; WBC white blood cell, Neu neutrophil, Lym lymphocyte, Mon monocyte, Eos eosinophil, RBC red blood cell, HGB hemoglobin, HCT hematocrit, MCV (fL) mean corpuscular volume, MCH mean corpuscular hemoglobin, MCHC mean corpuscular hemoglobin concentration, RDW-CV coefficient variation of red blood cell volume distribution width, RDW-SD (fL) standard deviation in red cell distribution width, PLT platelet, MPV (fL) mean platelet volume, PDW platelet distribution width, PCT thrombocytocrit. **f** Young (4–6 weeks of age, *n* = 10) and elder mice (12–13 weeks of age, *n* = 10) were treated with vehicle (0 mg/kg) or TKD at 50 mg/kg or 100 mg/kg every 3 days and their body weights were measured for statistics. **g** Pathological analysis of lungs, livers, spleens, and kidneys derived from the vehicle (TKD 0 mg/kg) or TKD-treated mice by using hematoxylin-eosin (H&E) staining. *n* = 5. Scale bars = 100 μm.

TKD induces KRAS degradation specifically and efficiently in cancer cells carrying different *KRAS* mutations and could be a potential anticancer drug candidate; thus, we explored the stability and toxicity of TKD before investigating its therapeutic effect. We first performed a stability analysis, which showed that the spatial structure of TKD remained intact at temperatures below 78.9 °C, indicating that TKD is quite stable under normal storage temperatures (Fig. 3a). Then, we incubated TKD in human serum samples at 37 °C for different times, and the results showed that the half-life of TKD in sera derived from either female or male donors was ~80 min (Fig. 3b and Supplementary Fig. S4a). We also collected the main organs of mice treated with TKD-Cy5 for further imaging analysis and found that TKD accumulation mainly occurred in the intestine and gallbladder, showing a typical metabolism process of protein drugs (Fig. 3c)^38^. Next, we evaluated the toxicity of TKD in mice. The acute toxicity test revealed that the highest tolerated dose of TKD in mice was 175 mg/kg for females and 150 mg/kg for males. Mice became listless when the dose of TKD was greater than 150 mg/kg, but the symptoms disappeared within a few hours (Fig. 3d and Supplementary Table S2). We thus chose 50 mg/kg and 100 mg/kg doses of TKD for further evaluation in mice. We first performed a blood test and observed that a short-term (1 h) treatment with 100 mg/kg TKD caused mild inflammation in a small number of mice (Fig. 3e). However, with long-term treatment, the body weight of both young and elderly mice remained unchanged compared to that of control mice (Fig. 3f). Moreover, histopathological analysis of organs such as the lung, liver, spleen, and kidney of TKD-treated mice showed that neither dose of TKD had apparent toxicity in mice (Fig. 3g). Therefore, our data demonstrate that the pharmacokinetics of TKD show a typical metabolic process of protein drugs and have no obvious side effects, indicating that TKD has good drug properties^39^.

### TKD suppresses the growth of cancer cells with different *KRAS* mutations

**Fig. 4:**
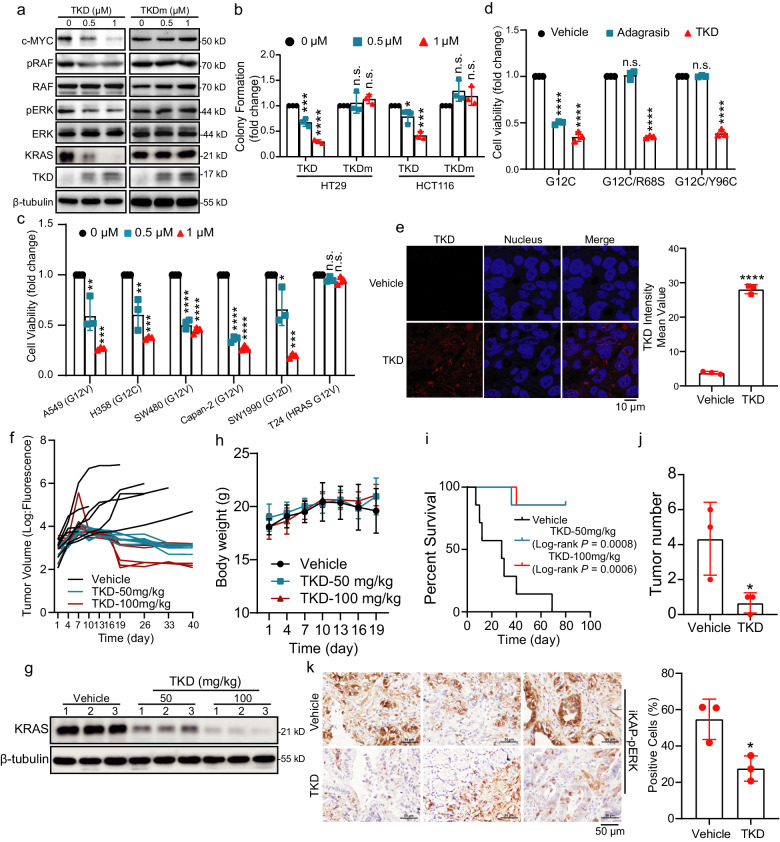
TKD suppresses growth of multiple cancers. **a** HCT116 cells were treated with TKD or TKDm for 24 h and the levels of the indicated proteins were evaluated by western blotting assay. **b** HT29 and HCT116 cells were treated with TKD or TKDm for 10 days for evaluating their colony formation ability. **c** Cancer cells with different *KRAS* or *HRAS* mutations were treated with the indicated amount of TKD for 3 days and cell viability was detected by CCK-8 assay. **d** H358 cells with or without *KRAS* R68S or Y96C mutation were treated with TKD (1 μM) or adagrasib (50 nM) for 48 h, and cell viability was detected by CCK-8 assays. **e** Mice with HCT116-derived subcutaneous tumors were treated with TKD (100 mg/kg) for 5 times every 3 days, and the distribution of TKD in the tumors was determined 2 h after the latest TKD treatment. Blue, nucleus; red, TKD. Scale bar = 10 μm. **f** HCT116-luc orthotopic transplantation mice were treated with vehicle (TKD 0 mg/kg) or TKD (50 or 100 mg/kg) every 3 days, and the tumor growth curve was analyzed using fluorescence statistic. *n* = 7. **g** Tumors collected from vehicle (TKD 0 mg/kg) and TKD-treated mice were lysed, and the expression of the indicated proteins was determined by western blotting assay. *n* = 3. **h** Mouse body weight was analyzed during TKD treatment. *n* = 7. **i** Survival status of mice with or without TKD treatment was analyzed. *n* = 7. **j** Tumor numbers of iKAP mice were analyzed. *n* = 3. **k** Positive phospho-ERK1/2 (Thr202/Tyr204) (pERK) cells in tumors of TKD-treated iKAP mice were determined using IHC. *n* = 3. Scale bar = 50 μm. Statistical analyses were performed using ordinary one-way ANOVA except for **j**, **k**, which were performed using unpaired *t*-test; Error bars, SD; n.s. not significant; *P < 0.05; **P < 0.01; ***P < 0.001; ****P < 0.0001.

We subsequently explored the potential anticancer activity of TKD. c-RAF (RAF), ERK and c-MYC are well-characterized downstream effectors of KRAS that are involved in promoting cancer cell proliferation^40^. We therefore evaluated the activation of the RAF/ERK/c-MYC signaling pathway in TKD- or TKDm-treated cancer cells and found that this pathway was significantly suppressed by TKD but not TKDm in HCT116 cells (Fig. 4a and Supplementary Fig. S4b). Colony formation assays showed that cell proliferation was inhibited by TKD but not by TKDm in cancer cells with different *KRAS* mutations (Fig. 4b). Further CCK-8 assays showed that TKD had a significant inhibitory effect on the growth of lung cancer cells, CRC cells and PDAC cells with different *KRAS* mutations, but had no inhibitory effect on HRAS-mutated cancer cells (Fig. 4c). Besides, we constructed two adagrasib-resistant cell lines that stably express R68S/Y96C mutant KRAS based on the H358 cell line (containing the *KRAS* G12C mutation)^14^. Then, we treated the cells with TKD and KRAS G12C-targeted drug adagrasib, respectively, and performed CCK-8 assays to determine the inhibitory effect of these agents on the drug-resistant cells. Data showed that the R68S/Y96C mutation significantly promoted the resistance of H358 cells to adagrasib, but TKD still maintained strong inhibitory effect on these drug-resistant cells (Fig. 4d).

To validate the permeability of TKD in tumor tissues, we generated a subcutaneous tumor model using HCT116 cells and administered TKD (100 mg/kg) intraperitoneally for 2 weeks. Two hours after the latest administration, the tumor tissues were collected for IF analysis, the data showed that TKD effectively infiltrated the tumor tissues, suggesting that TKD has a promising application for cancer therapy in vivo (Fig. 4e). We then constructed a cecal transplantation CRC model using HCT116-luc cells and treated the mice with TKD (50 mg/kg and 100 mg/kg) 7 times, and tracked tumor growth over an extended period and observed a significant suppression of tumor growth by TKD, accompanied by a decrease in KRAS expression in the tumors collected from the treated mice (Fig. 4f, g and Supplementary Fig. S4c). Importantly, there was no decrease in mouse weight during the treatment, and TKD-treated mice exhibited longer survival times than the control group (Fig. 4h, i). We also assessed the therapeutic effect of TKD in treating KRAS G12D transgenic iKAP mice, and our data demonstrated that TKD successfully suppressed tumor growth (Fig. 4j and Supplementary Fig. S4d). Then, we further evaluated the activation status of the downstream signaling of KRAS using immunohistochemical (IHC) analysis. Our results showed that the activity of downstream signaling pathways (indicated by pERK) was prohibited, indicating that TKD also has a good therapeutic effect on spontaneous tumors (Fig. 4k).

In addition, we compared the effect of TKD and the PROTAC LC-2 in degrading KRAS and suppressing the growth of cancer cells with different *KRAS* mutations. In contrast to TKD, LC-2 could only degrade the KRAS G12C mutant (Supplementary Fig. S4e)^22^. Moreover, TKD could prohibit the growth of either H358 (KRAS G12C)- or HCT116-derived tumors, whereas LC-2 could only prohibit H358-derived tumor growth (Supplementary Fig. S4f, g). In addition, TKD reduced KRAS expression in both HCT116 and H358 tumors, while LC-2 only reduced KRAS expression in H358 tumors (Supplementary Fig. S3h). Taken together, our results demonstrate that TKD induces KRAS degradation and suppresses the growth of cancer cells with different *KRAS* mutations and thus has a broader application than PROTACs.

### TKD sensitizes KRAS-mutant CRC to PD-1 antibody therapy

**Fig. 5:**
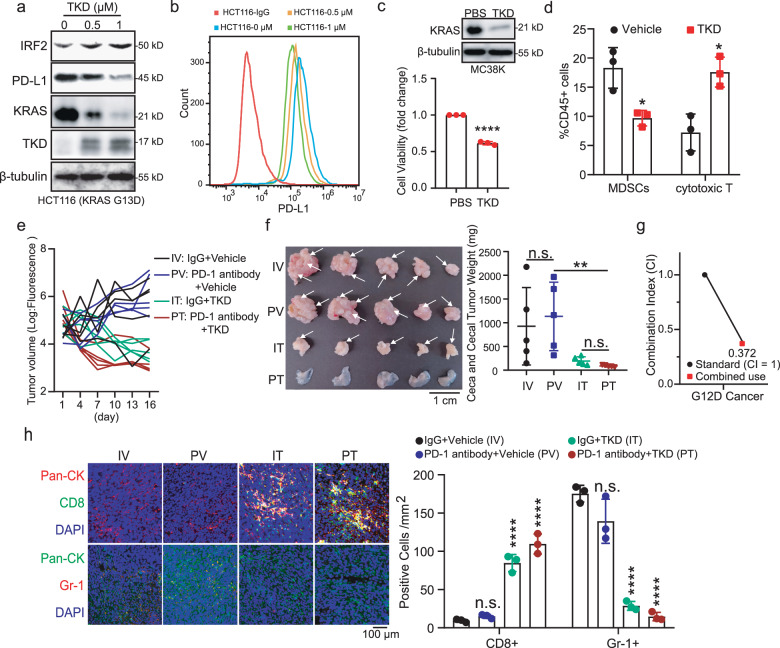
TKD restores immune microenvironment and improves ICB therapy. **a** HCT116 cells were treated with the indicated amount of TKD for 24 h and levels of IRF2, PD-L1, KRAS and β-tubulin were determined by western blotting. **b** HCT116 cells were treated with TKD for 24 h and the expression of PD-L1 on cell surface was determined by flow cytometry. **c** KRAS and β-tubulin were determined by western blotting assay and the cell growth was evaluated by CCK-8 assay in TKD-treated MC38K cells. **d** MC38K cecal transplantation CRC mice were treated with vehicle or TKD (50 mg/kg) per 3 days, then tumors were collected and dispersed into single cells for flow cytometry analysis, the amount of cytotoxic T cells (CD45^+^CD3^+^CD8^+^) and MDSCs (CD45^+^CD11b^+^Gr-1^+^) were analyzed. *n* = 3. **e** MC38K-luc cecal transplantation CRC mice were treated with vehicle, PD-1 antibody (200 μg), TKD (50 mg/kg), or the combination of TKD and PD-1 antibody every 3 days and tumor growth was recorded according to fluorescence intensity. *n* = 5. **f** Ceca, along with the cecal tumors were collected and weighed after 7 administrations as indicated. *n* = 5. Scale bar = 1 cm. Arrow, tumor site. **g** The synergistic effect of PD-1 antibody and TKD was analyzed by CI statistics. CI < 1 indicates synergistic effect. **h** IF staining for CD8^+^ T cells (CD8) and MDSCs (Gr-1) in TKD, PD-1 antibody or a combination of these two agents treated tumors. *n* = 3. Scale bar = 100 μm. Data are representative of three independent experiments. Statistical analyses were performed using unpaired *t*-test in **c** and **d**, Kruskal–Wallis test in **f**, and ordinary one-way ANOVA in **h**; Error bars, SD; n.s. not significant; *P < 0.05; **P < 0.01; ****P < 0.0001.

Previous studies have demonstrated that *KRAS* mutation inhibits the expression of interferon regulatory factor 2 (IRF2), a transcription factor that prohibits the migration of myeloid-derived suppressor cells (MDSCs) to the tumor microenvironment, and promotes the expression of programmed death-ligand 1 (PD-L1), thus creating an immunosuppressive microenvironment in tumors^7,41^. Considering that TKD significantly induces mutant KRAS degradation, we next explored the role of TKD in immune checkpoint blockade (ICB) therapy for CRC. Western blotting assays confirmed that KRAS degradation in TKD-treated cancer cells led to an increase in IRF2 and a decrease in PD-L1 in HCT116 cells (Fig. 5a and Supplementary Fig. S5a). The flow cytometry assay also demonstrated that PD-L1 expression on the cell surface was significantly reduced in TKD-treated cells (Fig. 5b). These results revealed that TKD could be used to reverse mutant KRAS-induced immune suppression. Then, we explored the activity of TKD in MC38K cells, a mouse CRC cell line stably expressing KRAS G12D. The data showed that KRAS was degraded and that cell growth was inhibited by TKD treatment (Fig. 5c and Supplementary Fig. S5b). Subsequently, we constructed a cecal transplantation CRC mouse model using MC38K-luc cells in C57BL/6 mice and treated the mice with TKD for a period of time. Flow cytometry analysis demonstrated that the number of cytotoxic T cells (CD45^+^CD3^+^CD8^+^) was increased and that of MDSCs (CD45^+^CD11b^+^Gr-1^+^) was decreased in TKD-treated tumors, indicating that TKD reverses the immune suppression in tumors driven by *KRAS* mutation (Fig. 5d). Then, we treated the mice with either TKD or PD-1 antibody alone or in combination. The results showed that tumor growth was prohibited in the combination treatment group (Fig. 5e). After treatment, the ceca were collected and weighed, and the data showed that the inhibitory effect of the PD-1 antibody and TKD combination was synergistic (indicated by combination index (CI) = 0.372) (Fig. 5f, g). Moreover, IF assays showed that cytotoxic T cells (CD8^+^) accumulated, whereas the number of MDSCs (Gr-1^+^) was decreased in tumors treated with combined PD-1 antibody and TKD (Fig. 5h). Importantly, severe CRC liver metastasis occurred in the control group and PD-1 antibody-treated group, but there was no cancer metastasis in the TKD-treated group and the combination group, indicating that TKD significantly suppressed CRC progression (Supplementary Fig. S5c). Together, our results demonstrate that TKD is a promising agent for restoring the tumor immune microenvironment and enhancing ICB therapy effects.

### The combination of TKD and cetuximab shows a good therapeutic effect in treating KRAS-mutant CRCs

**Fig. 6:**
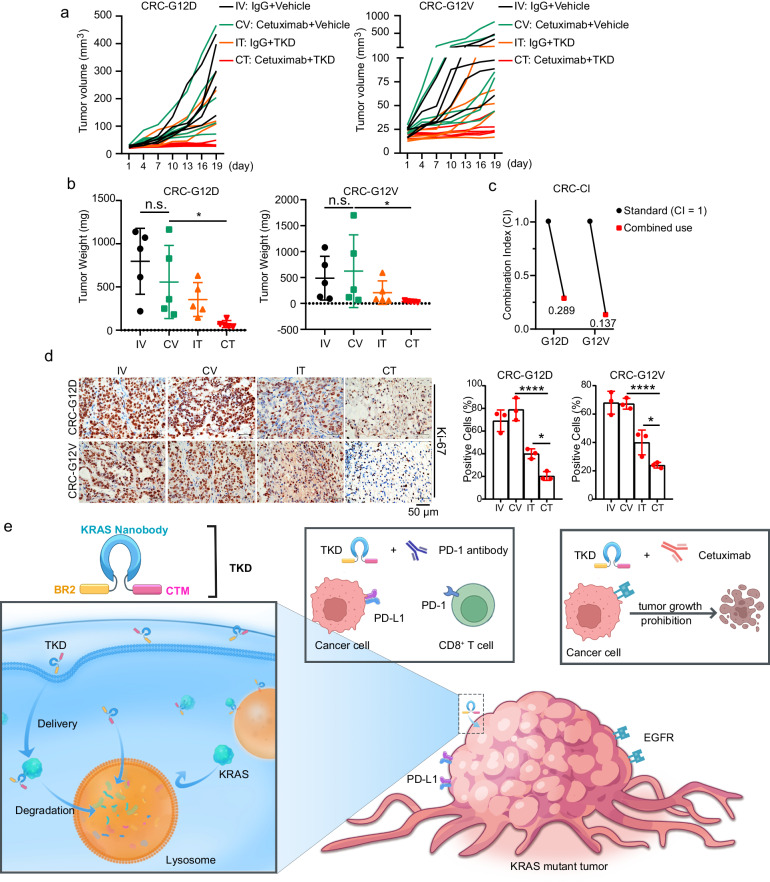
Combination of TKD and cetuximab in treating KRAS-mutant CRC PDX mouse models. **a** KRAS G12D and G12V mutant tumor tissues derived from CRC patients were transplanted subcutaneously into NSG mice, and the mice received 7 times administration of PBS + Vehicle, cetuximab (200 μg/mouse) + vehicle, PBS + TKD (50 mg/kg), and cetuximab (200 μg/mouse) + TKD (50 mg/kg). *n* = 5. **b** CRC tumor weight statistic. *n* = 5. **c** The combination effect of cetuximab and TKD was analyzed by CI statistics. CI < 1 indicates synergistic effect. **d** Analysis of Ki-67-positive cells using IHC in CRC tumors. *n* = 3. Scale bar = 50 μm. **e** TKD degrades KRAS and sensitizes KRAS-mutant cancers to PD-1 antibody and cetuximab. TKD recognizes and enters cancer cells through BR2 peptide, and binds to KRAS by nanobody, then the TKD–KRAS complex is recognized and degraded by lysosomes via CTM motif. The KRAS degradation induced by TKD reverses the resistance of KRAS-mutant cancers to immunotherapy and targeted therapy. Statistical analyses were performed using ordinary one-way ANOVA except for **b**, in which Kruskal–Wallis test was performed; Error bars, SD; n.s. not significant; *P < 0.05; ****P < 0.0001.

A recent study demonstrated the antitumor activity of combining a KRAS G12C inhibitor with the EGFR antibody cetuximab in KRAS G12C mutant CRC patients^42^. Besides, the proteomic analysis also indicated that TKD treatment could prohibit the activity of the EGFR signaling pathway (Fig. 2i). Therefore, we constructed patient-derived tumor xenograft (PDX) models using clinical CRC tissues containing different KRAS mutants and examined the combination of cetuximab and TKD in the KRAS-mutant CRC PDX models. The data showed that the growth of both KRAS G12D and G12V tumors was synergistically inhibited, with a CI value of 0.289 in G12D tumors and 0.137 in G12V tumors (Fig. 6a–c and Supplementary Fig. S5d). In addition, the number of Ki-67-positive cells was decreased in the cetuximab and TKD combination group compared with that in the cetuximab alone group in both G12D and G12V tumors (Fig. 6d). In summary, the results from PDX models suggest that TKD plays a promising role in the clinical therapy of KRAS-mutant CRCs.

## Discussion

KRAS became “druggable” due to the discovery of the binding pocket (S-IIP) in G12X (G12C/G12D) mutants. Several inhibitors have been developed to target this pocket and are currently undergoing clinical trials or have been approved for clinical therapy^43^. In addition to G12C and G12D mutants, most other KRAS mutants have also been found to contribute to cancer progression and drug resistance. It is worth noting that cancer cells have a tendency to develop secondary *KRAS* mutations as a means to counteract the effects of inhibitors such as sotorasib and adagrasib^44^. The pan-KRAS inhibitor reported recently prohibits KRAS activity by blocking the nucleotide exchange between KRAS-bound GTP and KRAS-bound GDP. This unique mechanism determines that it is not effective in prohibiting the activity of KRAS mutants with impaired GTP hydrolysis, such as G12R and Q61R/K/L^13^. Importantly, small molecules typically work by binding to specific amino acids, so unpredictable secondary mutations in these sites may lead to a higher risk of resistance; thus, TKD will show superior activity based on its unique nanobody-mediated target-binding mode. Unlike small molecules, antibodies bind to multiple amino acids on the surface of the target protein through complementary determining regions. For KRAS, the active site is embedded interiorly; thus, the mutations in the active region have little effect on antibody binding. Therefore, we constructed a pan-KRAS-targeting molecule termed TKD by fusing KRAS nanobodies with tumor-targeting CPPs and lysosome-binding motifs. TKD successfully degraded different KRAS mutants, including secondary mutations based on KRAS G12C, and prohibited the progression of KRAS-mutant cancers when used alone or in combination with other approved drugs (Fig. 6e). Our results showed that wild-type KRAS could also be degraded by TKD, but it is not necessary to worry about its toxicity to normal tissues, as TKD exhibits an ideal tumor-targeting capability.

The small size of the TKD contributes to its lower immunogenicity and improved tissue penetration. However, it also leads to a short half-life of TKD. Additionally, when TKD enters cells via endocytosis, some TKD molecules are recognized and degraded by lysosomes, resulting in only a portion of TKD being released into the cytoplasm to bind KRAS. Furthermore, the degradation of the KRAS–TKD complex leads to a gradual reduction in TKD within cells. As a result, continuous administration of TKD is necessary until the desired therapeutic effects are achieved. Our data suggest that regular dosing of TKD, either alone or in combination with other agents, can produce favorable therapeutic effects in KRAS-mutant cancers. Various chemical modifications, such as acylation and PEGylation, are available to enhance the stability of protein drugs, and some biological materials, such as hydrogels and lipid-based formulations, can also extend the half-life of protein drugs. Human serum albumin has a long half-life (~19 days); thus, the fusion of the albumin-binding domain and TKD may prolong the circulation time of TKD in the blood^45^. In addition, some peptides have been proven to effectively disrupt the endosome membrane and promote the release of contents, and the application of such a peptide in delivering proteins has shown remarkable success^46^; hence, the fusion of this type of peptide with TKD can also improve the efficiency of TKD delivery.

Although there have been many protein-based targeted drugs for cancer therapy, most of them still target oncoproteins on the membrane surface, such as human epidermal growth factor receptor 2 (HER2) and PD-1. Thus, in addition to the treatment of KRAS-mutant cancers, this study also provides a novel strategy for targeted drug development of intracellular oncoproteins. Theoretically, when the nanobody element of TKD is replaced with a nanobody targeting other intracellular proteins, the molecule will successfully degrade the target protein, similar to TKD. Therefore, this drug development strategy can make full use of the “undruggable” targets, especially those that have been demonstrated to play important roles in tumor progression, to design targeted drugs, which is of great significance for future cancer therapy.

## Materials and methods

### Cell lines and drugs

The HCT116, HT29, SW1116, RKO, and SW480 human CRC cell lines, the FHC normal colon epithelial cell line, the A549, NCI-H520 and H358 lung cancer cell lines, the T24 bladder cancer cell line, and the Capan-2 and SW1990 pancreas cancer cell lines were purchased from ATCC (Rockville, MD, USA). The MC38 mouse CRC cell line was provided by Prof. Wen-Ting Liao (Sun Yat-sen University Cancer Center), the MC38K cell line was derived from MC38 cells with KRAS G12D lentivirus. HT29 cells expressing KRAS Q61H, R68M, R68S, H95D, H95Q, H95R, Y96C, A59S, or A59T, and H358 cells expressing KRAS R68S and Y96C mutants were constructed using lentiviruses. Among these cells, HCT116, HT29, RKO, NCI-H520 and FHC cells were maintained in RPMI-1640 medium, SW1116, SW480 and SW1990 cells were maintained in L15 medium, and H358, T24, Capan-2, MC38, and MC38K cells were maintained in DMEM. All media were supplemented with 10% fetal bovine serum (FBS; Thermo Fisher Scientific, Waltham, MA, USA). Cells were cultured at 37 °C in 5% CO_2_ except for SW1116, SW480 and SW1990 cells, which were cultured at 37 °C without CO_2_ in the air mixture. An HCT116-GFP cell line was established by transfecting HCT116 cells with lentivirus stably expressing GFP. HCT116-luc and MC38K-luc cell lines were established by transfecting HCT116 and MC38K cells with lentivirus stably expressing luciferase. All cell lines were authenticated by short tandem repeat profiling and were tested for mycoplasma contamination. Lys05 (#S8369), cycloheximide (CHX, #S7418), bafilomycin A1 (Baf-A1, #S1413), MG132 (#S2619), adagrasib (#S8884) were purchased from Selleck Chemicals (Houston, TX, USA). Dimethyl sulfoxide (DMSO, #D4540) was purchased from Merck Life Science (St. Louis, MO, USA). LC-2 (#2502156-03-6) was purchased from Shanghai Biochempartner (Shanghai, China). PD-1 antibody and the IgG control were purchased from Bio-XCell (BE0146, clone RMP1-14; BE0089, clone 2A3).

### Western blotting assay

Briefly, cell pellets were suspended in RIPA lysis buffer (Beyotime Biotechnology, Shanghai, China) and centrifuged at 14,000× *g* for 30 min at 4 °C, and the supernatant was loaded onto a sodium dodecyl sulfate (SDS) polyacrylamide gel for electrophoresis, after which the proteins were subsequently transferred to polyvinylidene fluoride (PVDF) membranes (Millipore, Billerica, MA, USA). After blocking with 5% skim milk in Tris-buffered saline with Tween 20 (TBST), the membranes were incubated with primary antibodies followed by the corresponding horseradish peroxidase (HRP)-conjugated secondary antibody (TransGen Biotech, Beijing, China). Protein signals were detected using Clarity Western ECL Substrate (Beyotime Biotechnology) and visualized by using a ChemiDoc Imaging System (BioRad, CA, USA). The gray value was analyzed by using ImageJ software (National Institutes of Health). The primary antibodies used in this study included KRAS (#sc-30) that was purchased from Santa Cruz (Dallas, TX, USA); ERK (#67170-1-Ig), pERK (#80031-1-RR), C-RAF1 (RAF, #26863-1-AP), HRAS (#18295-1-AP), NRAS (#10724-1-AP), 6*His tag (#66005-1-Ig), BRAF (#20899-1-AP), GFP (#66002-1-Ig), and β-tubulin (#10094-1-AP) that were purchased from Proteintech (Chicago, IL, USA); PD-L1 (#13684s), pRAF (Ser338) (#9427t), Flag (#14793S), and c-MYC (#9402s) that were purchased from Cell Signaling Technology (Danvers, MA, USA); P62 (#ab109012) was purchased from Abcam (Cambridge, UK); LC3B I/II (#L7543) that was purchased from Sigma-Aldrich (Darmstadt, Germany), and IRF2 (#DF3093) that was purchased from Affinity Biosciences (Cincinnati, OH, USA).

### Alpaca immunization and screening of KRAS nanobodies

The KRAS nanobody was screened and generated by AlpaLife (KangTi Life Technology, Shenzhen, China). Briefly, an alpaca was immunized by using purified KRAS (1–169 aa) four times in two months. Then, the antibody sequences were amplified from the peripheral blood mononuclear cells to construct phage library. After two rounds of ELISA screening, the positive phages were collected and sequenced to obtain the sequences of the KRAS nanobody candidates.

### Purification of 6*His tag-fused proteins

Cancer cell-penetrating peptide BR2^31^, the nanobody against GFP^36^ or KRAS (1–169 aa), and the lysosome-binding peptide CTM^34^ or mutated CTM (CTM mt) were fused to construct the proteins GD, GDm, TKD and TKDm. The DNA sequences of these small proteins were synthesized and cloned and inserted into a 6*His-tagged pET28a (+) vector, and the plasmids were transformed into *E. coli* Rosetta cells for subsequent expression. The transformed bacteria were grown to an optical density at 600 nm (OD600) of ~0.8 at 37 °C, followed by induction with 0.5 mM isopropyl β-D-thiogalactopyranoside (IPTG) for 16 h at 20 °C and crushing using high pressure. Urea was added to the bacterial suspension at a final concentration of 8 M to fully denature the proteins, and then the suspension was centrifuged at 10,000 rpm for 30 min. The supernatant was collected and loaded onto a Ni-NTA resin (6*His-binding) column. The 6*His fusion proteins were then crudely purified according to the manufacturer’s instructions (Beyotime Biotechnology), and the target proteins were then verified by SDS-PAGE (Coomassie blue staining) and western blotting assay. Subsequently, the denatured protein was added into the prepared renaturation solution (1 M arginine monohydrochloride, 0.1 M Tris-base, 0.25 mM reduced glutathione, 0.25 mM oxidized glutathione) dropwise at a ratio of 1:100 and slowly mixed at 4 °C on a mixer overnight. The protein solution was rinsed and concentrated with phosphate-buffered saline (PBS) three times and then separated via IEC and HPLC. The impurities were discarded, and the target protein was collected for SDS-PAGE examination (Coomassie blue staining). Then, the target proteins were concentrated, and the endotoxin content was examined using End-point Chromogenic Tachypleus Amebocyte Lysate (BIOENDO, Xiamen, China) according to the manufacturer’s protocol. The absorbance of the reactants was measured at OD405 nm, and the endotoxin content was determined using a standard curve: *Y* = a*X + *b*, *r* > 0.96 (*Y* = OD405 value, *X* = endotoxin content). KRAS, as well as NTKD lacking the BR2 peptide, was purified in a manner that referred to TKD but did not require denaturation.

BR2 sequence: RAGLQFPVGRLLRRLLR;

GFP nanobody sequence: QVQLVESGGALVQPGGSLRLSCAASGFPVNRYSMRWYRQAPGKEREWVAGMSSAGDRSSYEDSVKGRFTISRDDARNTVYLQMNSLKPEDTAVYYCNVNVGFEYWGQGTQVTVS;

KRAS nanobody sequence: DVQLQESGGGLVQAGGSLRLSCVASGRTFSTYPTGWFRQAPGKEREFVARINLSGGITNYADSVKGRFTISRDNAKNTVYLQMNSLKPEDTAVYYCGGGSTTWAGGIPTNFDYWGQGTQVTVSSGR;

CTM sequence: KFERQKILDQRFFE;

CTM sequence mt: KFERAKILDARFFE.

### ELISA-based affinity constant measurement

Microtiter plates were coated with TKD, TKDm or IgG (mouse) protein overnight at 4 °C, blocked with 5% bovine serum albumin (BSA) in PBS for 1.5 h at room temperature (RT) and finally incubated with purified KRAS-His protein at a series of concentrations for 2 h at RT. The plate was then washed 5 times with PBS and incubated with anti-His (rabbit, Proteintech) primary antibody at RT for 2 h. After 5 washes, the plate was incubated with HRP-conjugated goat anti-rabbit antibody at RT for 1.5 h. Then, the plate was washed, the substrate tetramethyl-benzidine (TMB) was added to the wells, and the absorbance was measured at wavelengths of 450 nm and 600 nm using a SpectraMax M5 (Molecular Devices).

### BLI assay

The BLI assays were performed on a fortéBIO Octet Red96 instrument (Pall fortéBIO) at 30 °C as described previously^47^. Briefly, streptavidin (SA) biosensors (ForteBio) were preincubated in PBST (1× PBS buffer containing 0.1% Tween 20), a buffer used throughout the whole procedure, for 35 min. KRAS protein was chemically labeled by biotinylation with the EZ-link-Sulfo-NHS-biotin biotinylation kit (#A35358, Thermo Fisher, USA) followed by immobilization onto an SA-coated probe to explore KRAS−KDC binding events in PBST. Then, a 2-fold lower molar concentration of KDCs or purified HRAS/NRAS (#HY-P73919, #HY-P73741 MedChemExpress) was pumped through the biosensor for 100 s, followed by a 200-s disassociation and three rounds of regeneration with 10 mM glycine-HCl (pH 1.5). The kinetics curves were analyzed using ForteBio data analysis software. Raw curves aligned at association were adjusted with the baseline signals before the 1:1 binding model fitting was performed. Then, a global fit to all binding curves was conducted to calculate the overall kinetic parameters (*K*_D_, *K*_on_, *K*_dis_, etc.).

### NanoBiT assay and intracellular affinity estimation

NanoBiT assay was performed according to the manufacturer’s protocol (#N2014, Promega, Madison, USA). Briefly, all possible fusion gene forms of KRAS/TKD (nanobody) and luciferase subunits LgBiT/SmBiT were constructed, and the certain pair of plasmids were confirmed with the strongest luminescence signal after being co-transfected into 293 T cells and incubated with the substrate of luciferase. Then, the 293 T cells were co-transfected with the certain plasmids of different amounts at a ratio of KRAS:TKD or control plasmid at 1:1, and detect the luminescence signal after 24 h to draw luminescence intensity curve. Signal over 10 times higher than control was identified as specific protein–protein interaction, thus confirming the minimum transfection amount of KRAS or TKD (nanobody) plasmids. Subsequently, the increased intracellular protein concentration caused by the minimum plasmids’ transfection was measured and the affinity value of KRAS and TKD (nanobody) was estimated (assuming a plasmid transfection efficiency of 100% and the same expression level for KRAS and TKD).

### Co-IP and MS identification

The co-IP assays were performed using the IP kit with protein A + G magnetic beads (#P2179S, Beyotime Biotechnology) according to the manufacturer’s protocol. Briefly, after prewashing with IgG and protein A + G magnetic beads for 1 h at 4 °C, GD/GDm (1 μM)- or TKD/TKDm (1 μM)- and Lys05 (10 μM)-treated HCT116-GFP or HT29 and HCT116 cell lysates (containing 500 μg proteins) were incubated overnight with the IgG or 6*His tag antibody (#66005-1-Ig, Proteintech) at 4 °C followed by 4 h of incubation with protein A/G Sepharose beads. The beads were then washed three times with lysis buffer and eluted in 5× SDS-PAGE loading buffer for the immunoblot assay. A total of 2% input, namely, 10 μg of whole cell lysate, as well as 2% lysates that flow through the beads were used for immunoblotting, and the indicated protein was determined as a control. DDA mode MS was employed for the IP-MS analysis with an Orbitrap Fusion Lumos mass spectrometer equipped with EASY-nLC 1200 system (Thermo Fisher Scientific). Briefly, immunoprecipitated proteins were digested by trypsin using the filter-assisted sample preparation (FASP) method. Peptides were desalted and loaded on a nano trap column, and separated by an analytical column using a 120-min linear gradient at a flow rate of 600 nL/min. Subsequently, it was entered into the mass spectrometer for DDA data acquisition. Protein Discovery 2.4 software (Thermo Fisher Scientific) was used for DDA data search. We adopted the criteria for confident identification with a false discovery rate (FDR) < 0.01 at peptide and protein levels.

### Quantitative proteomics and bioinformatics analysis

TKD- or TKDm (1 μM)-treated HCT116 cells were dissolved in cell lysis buffer. After trypsinization using the FASP method, peptides were vacuum lyophilized. The iRT-Standard (Biognosys, MA, USA) was added into each sample and data-independent acquisition (DIA) MS were collected by Orbitrap Fusion Lumos mass spectrometer. The DIA raw data were processed and searched by the direct DIA module of Spectronaut 18 (Omicsolution Co., Ltd, Shanghai, China). The identification was performed using a 0.01 *Q*-value (adjust *P* value) cutoff on precursor and protein levels. The identification FDR threshold at PSM, peptide, and protein levels was 0.01. All the data were searched against UniProt Human protein database (http://www.uniprot.org). DIA data analysis was performed using Limma software for normalized and scaled data and differential expression analysis. The GO analysis was determined using the database for annotation, visualization, and integrated discovery (DAVID) functional annotation tools.

### IF assay

HCT116-GFP cells were fixed in 4% paraformaldehyde and permeabilized with 0.1% Triton X-100 for 30 min before being blocked with 5% BSA for 2 h at RT. The cells were then incubated with primary antibody against LAMP2 for 2 h at RT before being washed with 1% TBST 3 times for 3 min each time (#66301-1-Ig, Proteintech). The cells were subsequently incubated with Alexa Fluor 555-labeled donkey anti-mouse IgG antibody for 1.5 h at RT before being counterstained with DAPI (Thermo Fisher Scientific, Waltham, MA, USA) and observed by laser scanning confocal microscopy (Carl Zeiss AG, Jena, Germany). For tissues, paraffin-embedded samples were sectioned at 4-μm thickness. Antigen retrieval was performed in a pressure cooker at 95 °C for 10 min in citrate antigen retrieval solution (P0081, Beyotime). Sections were then blocked in PBS containing 2% goat serum albumin for 2 h at RT. Then, the slides were incubated in a mixture of antibodies overnight at 4 °C. Except for 6*His-tag, the following primary antibodies were used: rat anti-Gr-1 (#108401, BioLegend), mouse anti-Cytokeratin Pan (#ab7753, Abcam), and rabbit anti-CD8 (#bs-0648R, Bioss). The slides were washed with cold PBS and incubated with a mixture of two secondary antibodies raised in different species for 2 h at RT in the dark. The following secondary antibodies were used: Alexa Fluor 488-labeled anti-rabbit (A11008, Life Technologies), Alexa Fluor 594-labeled anti-rat (A11007, Life Technologies), Alexa Fluor 488-labeled anti-mouse (A11001, Life Technologies), and Alexa Fluor 594-labeled anti-mouse (A21203, Life Technologies). Slides were counterstained with Antifade Mounting Medium with DAPI (P0131, Beyotime) and examined by fluorescence microscopy.

### CCK-8 and colony formation assay

Cell viability was measured using a CCK-8 Cell Counting Kit (Beyotime Biotechnology, Shanghai, China). A total of 1500 cells were seeded in 96-well plates and treated with drugs at various concentrations for multiple durations. CCK-8 reagent was added, and the cells were incubated at 37 °C for 2 h. For the colony formation assay, cells were seeded in 12-well plates at a density of 500 cells per well. After 10 days, the cells were fixed with methanol and stained with 0.1% crystal violet. The number of colonies was then quantified for analysis.

### Cy5 labeling of NTKD and TKD

NTKD and TKD were labeled with Cy5 with an EZLabel™ protein Cy5 labeling kit (BioVision, Milpitas, CA, USA) according to the manufacturer’s protocol. Briefly, Cy5 powder was dissolved in DMSO, mixed with NTKD or TKD protein, and then incubated on a rotary shaker for 1 h at RT. After washing the resin in a spin column, the labeling reaction mixture was loaded into a column and centrifuged for 2 min at 1500× *g* to collect the eluant. The eluant included Cy5-labeled NTKD or TKD (NTKD-Cy5, TKD-Cy5).

### Flow cytometric analysis

FHC, HT29 and HCT116 cells were treated with 0.5 μM TKD-Cy5, NTKD-Cy5, or PBS for 2 h in RPMI-1640 complete medium. The cells were then collected and analyzed on a CytoFLEX LX flow cytometer (Beckman Coulter, CA, USA). For tissues, the vehicle- or TKD-treated MC38K cecal tumors were digested into single cells using the KeyGEN tissue dissociation Kit (KGA829, KeyGEN BioTECH) following a standard protocol. Digested tumors were mashed through 40-μm filters into RPMI-1640 and centrifuged at 300× *g* for 5 min at 4 °C. Then, the cells were blocked with PBS containing 5% BSA for 10 min and incubated with surface antibody mix for 2 h at RT. Antibodies against CD45 (APC-Cy7, #557659, BD Biosciences), CD3 (PE, #100206, BioLegend), CD8a (Alexa Fluor 700, #100730, BioLegend), CD11b (PE, #101208, BioLegend) and Gr-1 (FITC, #108405, BioLegend) were used.

### IHC analysis

Tumor xenograft sections were deparaffinized in xylene, rehydrated in a graded series of ethanol solutions and processed for IHC. After antigen retrieval and blocking with goat serum, the slides were incubated with a pERK or Ki-67 antibody overnight at 4 °C, washed with PBS, and incubated with the appropriate peroxidase-conjugated secondary antibody. 3,3’-Diaminobenzidine served as a chromogen for visualized immunostaining, and the sections were counterstained with hematoxylin. Stained cells were evaluated by two independent investigators according to the following scale (0–2): high expression corresponded to a staining score of 2, low expression corresponded to a staining score of 1, negative expression corresponded to a staining score of 0, and positive cells were indicated by high expression.

### Tumorigenicity and imaging in mice

Four- to six-week-old BALB/c nude mice and C57BL/6 mice were purchased from Guangdong Medical Laboratory Animal Center (Guangzhou, China). NSG mice were purchased from Shanghai Model Organisms Center (Shanghai, China). All mice were maintained under standard conditions and treated according to institutional guidelines for animal care. For BALB/c nude mice, 3 × 10^5^ HCT116 or H358 cells were suspended in a 1:1 mixture of PBS:Matrigel and subcutaneously injected into the flanks of the mice, and 1 × 10^6^ HCT116-luc cells were suspended in PBS and injected into the cecal serosa. Besides, 2 × 10^6^ MC38K-luc cells were suspended in PBS and injected into the cecal serosa of C57BL/6 mice. For the PDX mouse model, clinical KRAS G12D and G12V CRC tissues were evenly divided into 2 mm × 2 mm × 2 mm pieces and subcutaneously embedded into the flanks of NSG mice. When the tumors reached ~5 mm in diameter or the fluorescence intensity reached ~3 × 10^3^ in the tumor region, the mice were randomized into treatment and control groups. The treatment group received oral gavage of LC-2 (50 mg/kg) or intraperitoneal administration of TKD (50 mg/kg or 100 mg/kg), PD-1 antibody (200 μg) or cetuximab (200 μg) every three days, whereas the control groups received vehicle (PBS, DMSO, IgG, or their mixtures). An additional treatment group was given PD-1 antibody (200 μg) combined with TKD (50 mg/kg) or cetuximab (200 μg) combined with TKD (50 mg/kg). The body weight of the mice was monitored every three days during the experiments to evaluate overall health. The iKAP CRC mouse model (*Villin-cre-ERT;Apc*^*lox/lox*^*;Trp53*^*lox/lox*^*;tet-O-LSL-KRAS*^*G12D*^) was kindly provided by Prof. Wen-Ting Liao (Sun Yat-sen University Cancer Center). To induce colorectal tumors in iKAP, 4-OHT (1 mg/mL) was applied via cecal injection, followed by Dox chow (200 mg/kg) at 8 weeks of age. For treatment in the iKAP model, iKAP mice with ~4 mm tumors were randomly grouped into two cohorts, and doxycycline (Dox) chow was replaced with Dox water. iKAP mice were treated with PBS and TKD every three days.

For acute toxicity test, BALB/c nude mice were gradually intraperitoneally administered 250 mg/kg, 200 mg/kg, 175 mg/kg, 150 mg/kg, 100 mg/kg, 50 mg/kg, 25 mg/kg, or 0 mg/kg (PBS) TKD, and the survival and status of the mice were recorded. All mice were euthanized at the end of the study, and tumors, lungs, livers, spleens, and kidneys were collected for further analysis.

For in vivo imaging, TKD-Cy5 (60 μM), NTKD-Cy5 (60 μM) or Cy5 control (60 μM) was injected into mice burdened with or without xenograft tumors through the tail vein (100 μL/mouse), and the fluorescence intensity of the mice or main organs, such as the heart, liver, spleen, lung, kidney, intestine, and gallbladder, was detected and analyzed with an IVIS Spectrum imaging system (Perkin Elmer, MA, USA). All animal experiments were approved by The Institutional Animal Care and Use Committee at Sun Yat-sen Cancer Center (L025501202108036).

### Clinical samples

Human sera and clinical tumor tissues were obtained from the Sun Yat-sen University Cancer Center Department of Pathology, and the experiments were approved by the Ethics Committee of Sun Yat-sen University Cancer Center (GZR2020-052).

### Statistical analysis

Statistical analyses were performed using GraphPad Prism 8. The CI was analyzed with the formula CI = AB/(A × B), among which A represents the tumor weight ratio of the drug A treatment group and vehicle group, B represents the tumor weight ratio of the B drug treatment group and vehicle group, AB represents the tumor weight ratio of the drug A and B co-treatment group and vehicle group, and a synergistic effect is indicated by CI < 1. Experiments were performed with 3 biological replicates, and the data from 3 independent experiments are presented as the means ± SD and were compared using unpaired *t*-test (groups ≤ 2) or ordinary one-way ANOVA (groups ≥ 3). Data that were not Gaussian distribution were analyzed using the Kruskal‒Wallis test, and data with two independent variables were analyzed using two-way ANOVA. If any mice died during treatment, the overall tumor growth data were analyzed using mixed-effects analysis.

### Supplementary information

Supplementary information

## Data Availability

The raw data of this study has been uploaded onto the Research Data Deposit public platform (www.researchdata.org.cn), with the approval number as RDDB2024683824. Other materials are available upon request from the authors.
