# Supplementary material for: A pan-KRAS degrader for the treatment of KRAS-mutant cancers
Source: Cell Discov. 2024 Jun 28;10:70. doi: 10.1038/s41421-024-00699-4 (PMC11211324; doi:10.1038/s41421-024-00699-4)
Supplement: Supplementary file 1 — Supplementary information [file 41421_2024_699_MOESM1_ESM.pdf]

Supplementary information

Fig. S1

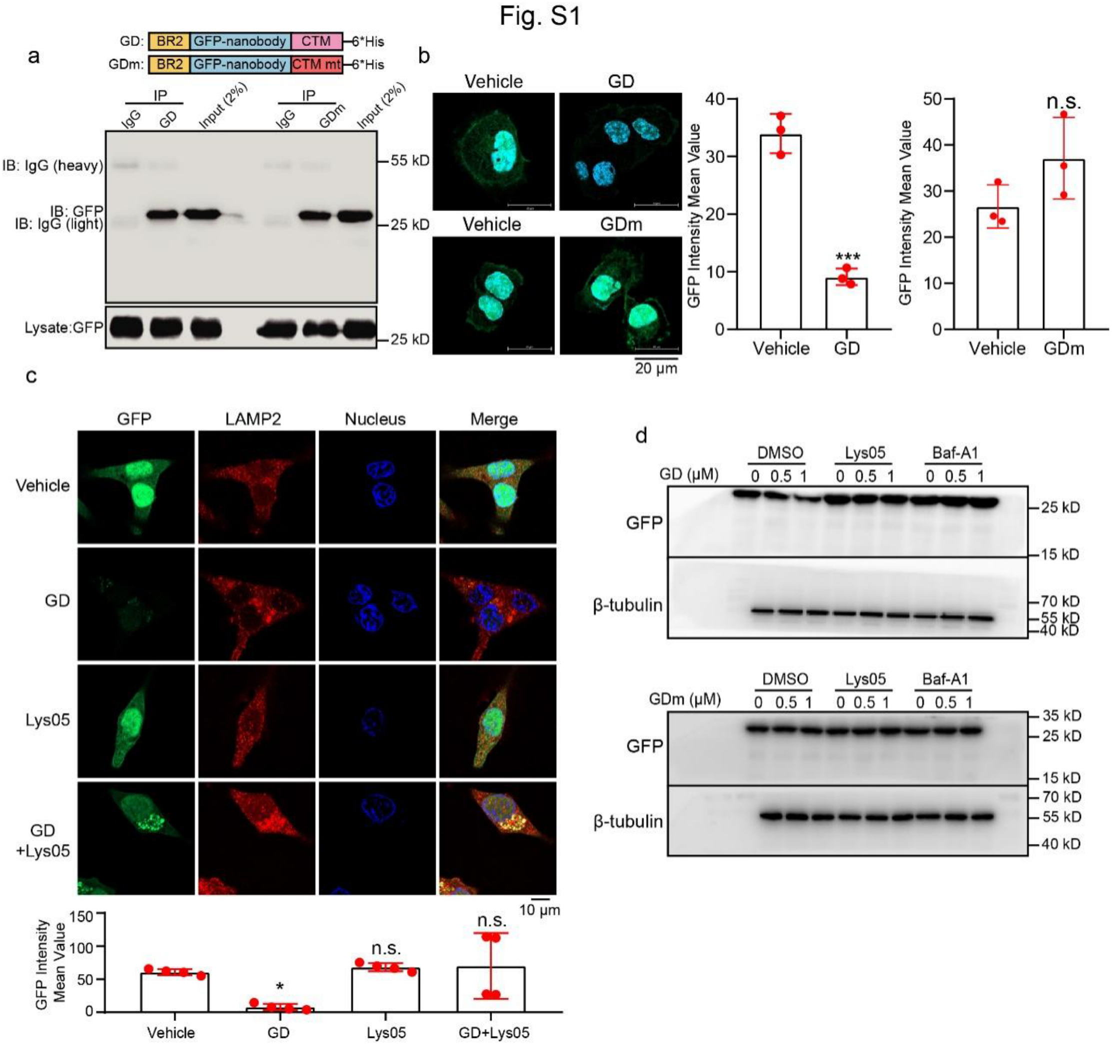

Supplementary Fig. S1 Determination of GD in degrading GFP. a HCT116-GFP cells were treated with GD or GDm (10 μg/mL), the cell lysates were immunoprecipitated with a 6*His-tag antibody, and the bound proteins were analyzed by immunoblotting with a GFP antibody. The GFP level in the whole-cell lysate were used as a loading control. Top, diagram of GD and GDm; bottom, western blotting of the co-IP assay. **b** HCT116-GFP cells were treated with GD or GDm (0.5 μM), and the green fluorescence of the cells was detected and analyzed. Scale bar = 20 μm. **c** Immunofluorescence analysis of the localization of GFP in the HCT116-GFP cells treated with GD and Lys05. Red, LAMP2; green, GFP; blue, nucleus (DAPI); arrow, colocalization of GFP and lysosomes. Scale bar = 10 μm. **d** HCT116-GFP cells were treated with either GD or GDm alone, or together with Lys05 (10 μM), or Baf-A1 (100 nM) and the expression of indicated proteins was examined by western blotting assay. Statistical analyses were performed using unpaired *t*-test except for (**c**), which was performed using one-way ANOVA; Error bars, SD; n.s., not significant; *$P < 0.05$; ***$P < 0.001$.

Fig. S2

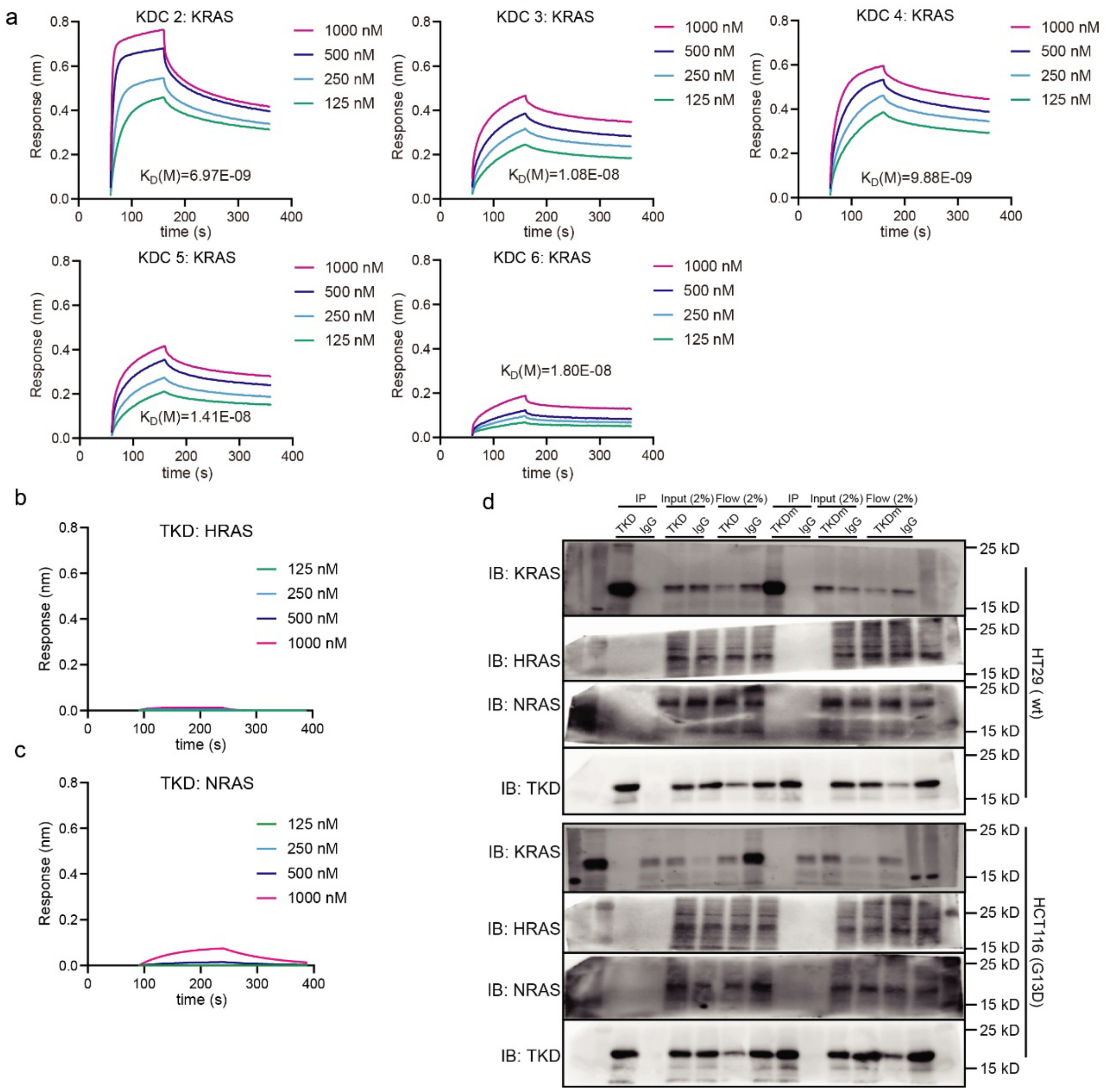

Supplementary Fig. S2 Verification of the affinity of KDC to KRAS. a BLI assays were performed with purified KRAS and KDCs (KDC 2-6) that constructed with the nanobody of positive phages. The concentrations of KDCs flowing over the KRAS-coated probes as indicated. **b**-**c** BLI assays were performed with purified TKD and HRAS (**b**) or NRAS (**c**). The concentrations of HRAS or NRAS flowing over the TKD-coated probes as indicated. **d** HT29 and HCT116 cells were treated with a combination of TKD or TKDm (0.5 μM) and Lys05 (10 μM) for 6 h and IP assays were performed by using a 6*His-tag antibody. The levels of KRAS/HRAS/NRAS and TKD-His in the immunoprecipitates, the whole cell lysates (2% Input), as well as lysates flow through the beads (2% Flow), were detected by western blotting assay, and the ratio of precipitated KRAS in total KRAS was analyzed.

Fig. S3

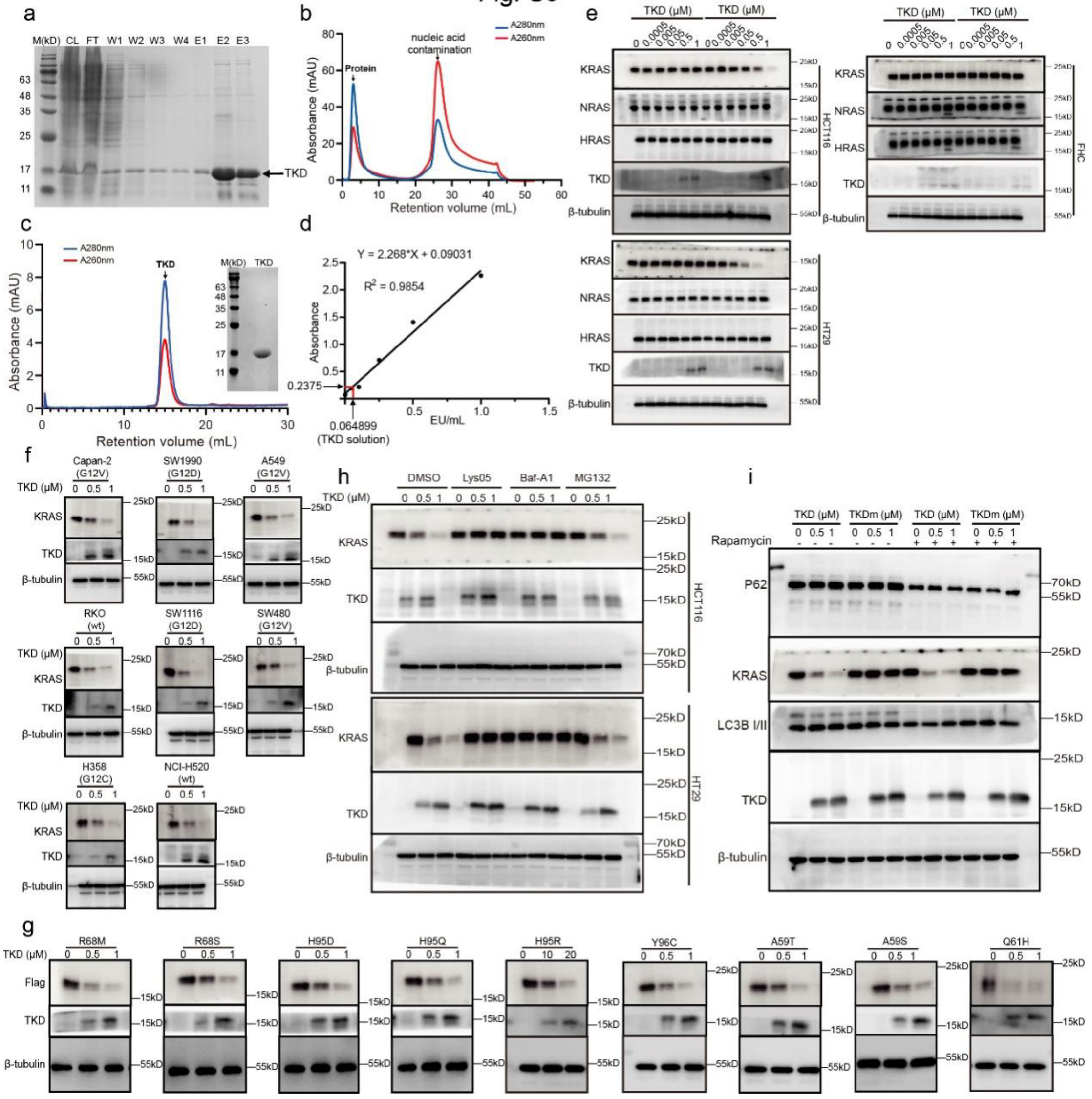

Supplementary Fig. S3 Purification of TKD and its effect in degrading KRAS. a Proteins were denatured and purified with 6*His Ni-NTA resin and determined by Coomassie blue staining. CL, cell lysate; FT, flow through; W1-W4, washing solution; E1-E3, elution. **b** Renatured proteins were purified using IEC. Proteins were indicated by and collected at high A280 absorbance (blue line). The eluted nucleic acids were indicated by A260 absorbance (red line). X axis, retention volume; Y axis, absorbance. **c** The protein solution from IEC was further purified using HPLC and the target protein TKD was examined by Coomassie blue staining. The impurities with molecular weight differ from TKD were removed. **d** Endotoxin examination of purified TKD. X axis, endotoxin content; Y axis, absorbance value of sample at OD.405. **e** HT29, HCT116, and FHC cells were treated with TKD or TKDm at a series of concentrations as indicated for 24 h, and the expression of KRAS, HRAS, NRAS, TKD-His, and β-tubulin was detected. **f** Cancer cells with different KRAS mutants as indicated were treated with TKD for 24 h. The expression of KRAS, TKD-His, and β-tubulin was detected. **g** HT29 cells stably expressing KRAS G12C combined with the secondary mutations as indicated were treated with TKD for 24 h. The expression of Flag, TKD-His, and β-tubulin was detected.

h HT29 and HCT116 cells were treated with TKD alone or together with Lys05 (10 μM), Baf-A1 (100 nM), MG132 (1 μM) for 24 h. The expression of KRAS, TKD-His, and β-tubulin was detected. **i** H358 cells were treated with TKD alone or a combination of TKD and rapamycin (25 μM) for 24 h, then cells were lysed and the expression of LC3B I/II, P62, KRAS, TKD-His, and β-tubulin was determined using western blotting assays.

Fig. S4

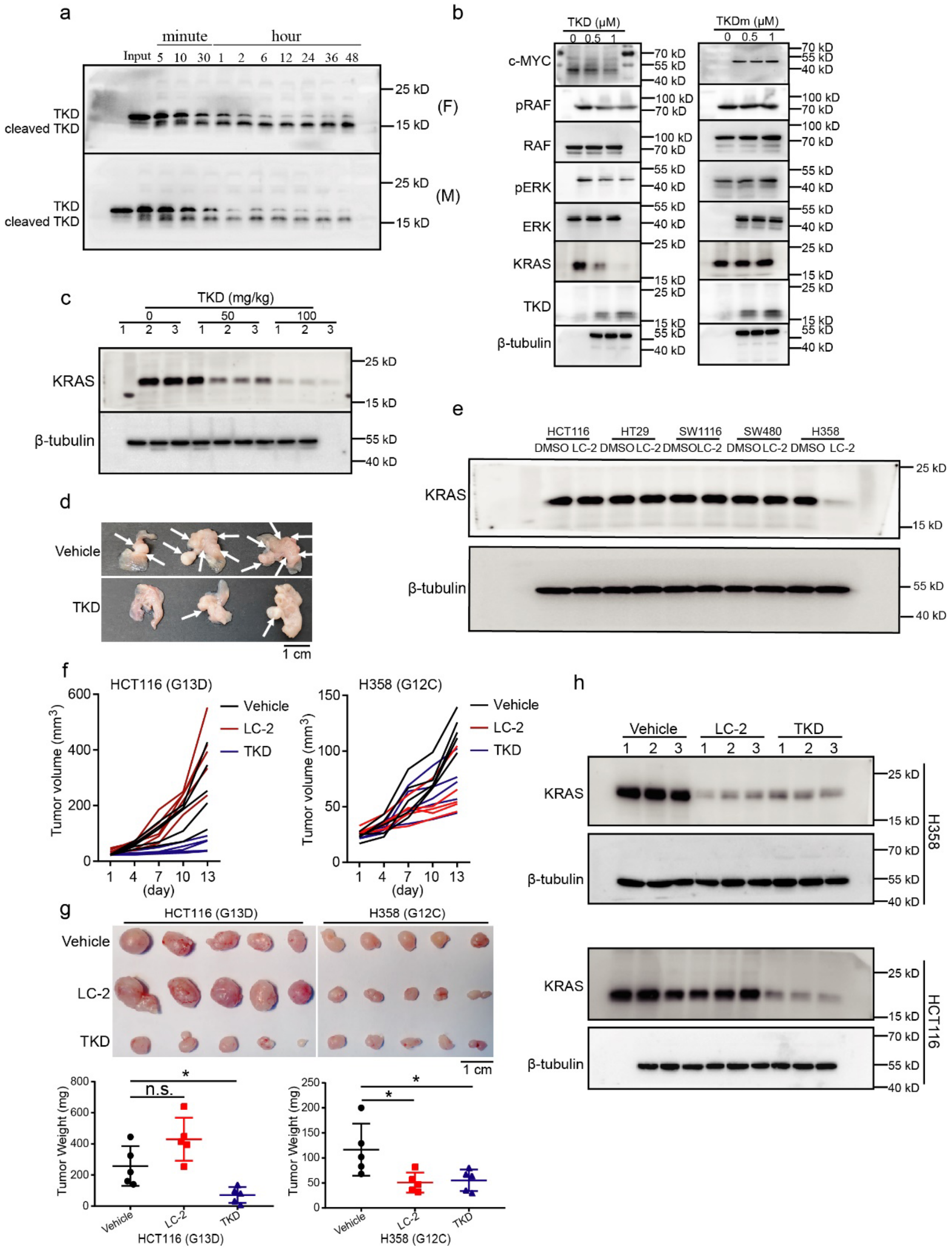

Supplementary Fig. S4 TKD induces KRAS degradation and tumor growth suppression, has a broader application than PROTAC-LC2. a 0.1 µg purified TKD was mixed with 27 µL of human sera and incubated at 37℃ for different durations as indicated, the degradation of TKD was evaluated by western blotting. Input, purified TKD; F, sera derived from the female; M, sera derived from the male. **b** HCT116 cells were treated with TKD or TKDm for 24 hours and the levels of indicated proteins were evaluated by western blotting. **c** Tumors collected from vehicle (TKD 0 mg/kg) and TKD-treated mice were lysed, and the expression of indicated proteins was determined by western blotting assay. *n* = 3. **d** Ceca tissues derived from iKAP mice with or without TKD treatment. Arrow, tumor. Scale bar = 1 cm. **e** Verification of the effect of LC-2 on promoting KRAS degradation in cells with different KRAS status by using western blotting. Data are representative of three independent experiments. **f-g** Nude mice bearing HCT116- or H358-derived tumors were treated with vehicle, TKD (50 mg/kg) or LC-2 (50 mg/kg) every 3 days, the tumor volume was recorded (**f**), tumor pictures were taken and the tumor weight was analyzed (**g**) after 5 times administration. *n* = 5. Scale bar = 1 cm. (**h**) Above HCT116- and H358-derived tumors were lysed, and the expression of indicated proteins was determined by western blotting. n = 3. Statistical analyses were performed using ordinary one-way ANOVA; Error bars, SD; n.s., not significant; * *P* <0.05.

Fig. S5

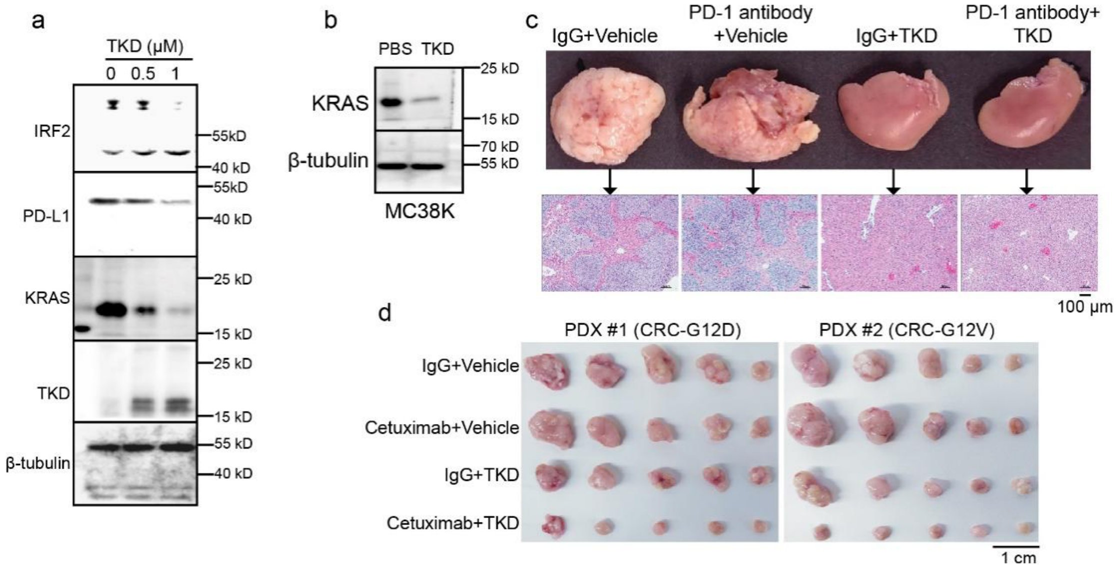

Supplementary Fig. S5 Role of TKD in cancer immunotherapy and targeted therapy. a HCT116 cells were treated with the indicated amount of TKD for 24 h and levels of IRF2, PD-L1, KRAS and β-tubulin were determined by western blotting assay. **b** KRAS and β-tubulin were determined by western blotting in TKD treated MC38K cells. **c** Mice livers were collected for H&E staining. *n* = 3. Scale bar = 100 µm. **d** KRAS G12D and G12V mutant tumor tissues derived from CRC patients were transplanted subcutaneously in NSG mice, respectively, and the mice received 7 times administration of PBS + Vehicle, cetuximab (200 µg/mouse) + vehicle, PBS + TKD (50 mg/kg), and cetuximab (200 µg/mouse) + TKD (50 mg/kg). *n* = 5. Scale bar= 1 cm.

Supplementary Table S1: Nanobodies screened by ELISA.

No.	Nanobody Sequence	ELISA-1st		ELISA-2nd	
		Ag	BSA	Ag	BSA
1	DVQLQESGGGLVQAGGSLRLSCVASGRTFSTYPTGWFRQAPGKEREFVARINLSGGITNYADSVKGRFTISRDNAKNTVYLQMNSLKPEDTAVYYCGGGSTTWAGGIPTNFDYWGQGTQVTVSSGR	0.3630	0.0493	0.2924	0.0588
2	DVQLQESGGGLVQAGGSLRLSCVAPARTVSTYPTGWFRRAPGKEREFVARINFSGGITNYADSVKGRFTISRNNTKNTVYLQMNSLKPEDTAVYYCGGGSTTWADGIPINFDYWGQGTQVTVSSGR	0.3330	0.0555	0.3525	0.0547
3	DVQLQESGGGLVQPGGSLRLNCAASGFVFSDYAMSWVRQAPGKGLEWVSAIYGGGYNPIYTDSVKGRFTISRDNAKNTLYLQIRTLKPEDTGVYYCAKEQTFGTFLNDTYYSPSYYDWGQGTQVTVSSGR	0.2861	0.0492	0.2473	0.0661
4	DVQLQESGGGLVQPGGSLRLSCAASGFTFGNTGMSWVRQAPGKGLEWVSGISSGGAFSFYADSVKGRFTGSDNSAKNTVYLQMNSLQPEDTGVYYCAAGPRVALLTTPWTNPQSYHYWGQGTQVTVSSGR	0.2373	0.0495	0.1707	0.0454
5	DVQLQESGGGLVQPGGSLRLNCAASGFVFSDYAMSWVRQAPGKGLEWVSAIYGGGYNPIYADSVKGRFTISRDNAKNTLYLQIRTLKPEDTGVYYCAKEQTFGTFLNDTYYSPSYYDWGQGTQVTVSSGR	0.2296	0.0512	0.2023	0.0731

6	DVQLQESGGGLVEPGGSLRLSCAASGSIFGL DVMGWYRQAPGKQRELVAIITRGGTANYA DSGKGRFTISSDYAKKTVSLQMGSLIPEDTAV YYCSALVLGTNYWGQGTQVTVSSGR	0.208 0	0.049 9	0.152 8	0.045 8

Abbreviation: Ag, antigen (KRAS protein); BSA, bull serum albumin.

Supplementary Table S2: Acute toxicity test of TKD in mice.

TKD (mg/kg)	Status
250	Death
200	Death
175	Survive, twitch for 2 h, listlessness and recovered 8 h after administration
150	Survive, listlessness and recovered 1-2 h after administration
100	Survive, no abnormalities
50	Survive, no abnormalities
25	Survive, no abnormalities
0 (vehicle)	Survive, no abnormalities
